# Self-regulation and performance among elite youth soccer players: the role of approach-avoidance motivation

**DOI:** 10.3389/fpsyg.2024.1416931

**Published:** 2024-10-14

**Authors:** Mounir Hamoud, Stig Arve Sæther, Gunnar Bjørnebekk

**Affiliations:** ^1^Department of Education, University of Oslo, Oslo, Norway; ^2^Department of Sociology and Political Science, Norwegian University of Science and Technology, Trondheim, Norway; ^3^Department of Special Needs Education, University of Oslo, Oslo, Norway

**Keywords:** self-regulation, performance, elite youth soccer players, approach-avoidance motivation, achievement goals, fear of failure, achievement motives

## Abstract

**Introduction and methods:**

This study aimed to investigate the motivational processes behind self-regulated learning and performance among 192 soccer players (82 girls) for three age groups (14–16 years old) eligible for the Norwegian national football team. A conditional process model was proposed and tested with achievement goals as mediators between achievement motives on the one hand and coach-reported performance and self-regulated learning on the other hand. The probability of success was examined as a potential moderator in the motivational process.

**Results:**

As predicted, motives to achieve success directly influenced planning and reflection/evaluation, whereas the influence of the success motive on regulation of effort was explained partly by task- and self-based approach goals. The motive to achieve success was, however, particularly crucial for maintaining these beneficial regulatory processes when the probability of success was found to be from moderate to low. Concerning the avoidance paths, the data supported only some of our original hypotheses. The motive to avoid failure predicted all three types of avoidance-based (task, self, and other) and other-based approach goals but did not contribute to explaining planning, reflection/evaluation, regulation of effort, or performance. There were no significant correlations between motivation variables and coach-reported performance. Moreover, girls were more motivated to avoid failure than boys, while both sexes achieved similar scores for football-specific self-regulated learning, probability of success, achievement goals, and motive to achieve success.

**Discussion:**

The results are discussed considering a hierarchical motivation model.

## Introduction

The prioritization of talent development in soccer has contributed to increasing scientific interest in both identifying and developing soccer talents over the past 20 years (e.g., Gledhill et al., 2017; Williams et al., 2020). Developing high level of soccer skills is a complex process based on multidimensional global and soccer-specific factors, including physiological, sociological, psychological, physical, and technical soccer-based factors (Williams et al., 2020). In this multifaceted spectrum, there has been increasing interest in the psychological dimension, such as motivation (Musculus and Lobinger, 2018) and expertise development (McCardle et al., 2018), even though the significance of motivation in talent development and soccer performance is well documented (Gledhill et al., 2017).

Elliot’s (1999) hierarchical motivation model has been widely used in educational and sport studies and has been shown to predict learning processes and future achievements. The hierarchical model operates with achievement goals as the construct designed to help us understand the direction of behavior by addressing the question of “what” individuals want to achieve. It also incorporates achievement motives to account for the energization of behavior, addressing the question of why individuals want to achieve (“the antecedent”) (Elliot and Thrash, 2001). For young football talents to transition into elite football, not only is energy and goal direction required but also the ability to regulate their own learning processes (Larsen et al., 2012). Consequently, how motives and goals influence the way a group of talents regulates their own learning processes is likely to be crucial for the learning outcomes and performances over time, making it a compelling subject for further investigation. However, few studies include both the antecedents and the outcomes simultaneously (Lochbaum et al., 2017), and the links in the hierarchical model have not been similarly explored in soccer studies (Lochbaum and Gottardy, 2015). In the present research, we will use the hierarchical model as a framework to investigate motivational processes (both what provides energy and direction) behind self-regulated learning and performance among talented soccer players.

## A hierarchical motivation model

In Elliot’s (1999) model, the achievement motives are presumed to energize behavior. However, specific guidelines for achieving what behavior has been activated have not been provided. Therefore, they are not directly related to outcomes, such as self-regulated learning or performance. Their function is a general motivating factor that produces achievement outcomes through more specific goals. Moreover, motivation is not only created by the achievement motives—situational factors must also be considered. An important message from the classical motivation tradition was precisely that individuals that are motivated by success (High Ms) and those motivated by fear of failure (High Mf) would be quite differently motivated in different achievement situation (Atkinson, 1964; Gjesme, 1981). Activated motivation is, for example, assumed to be very different when the task they must deal with is easy, moderate, or very difficult (e.g., Atkinson, 1964; Nygård, 1977).

### Achievement motives

In research on achievement motivation, two primary motives are usually applied: *desire to achieve success* and *fear of failure*. The motives are relatively stable personality characteristic in terms of a capacity to anticipate affect in achievement situations. The motive to achieve success (Ms) is defined as the capacity to expect a pleasant emotional state in challenging situations. Conversely, the motive to avoid failure (Mf) is defined as the capacity to expect unpleasant affective changes to occur when the outcome of a mastery attempt is uncertain (Gjesme and Nygård, 1970). These are situations where both success and failure are possible outcomes (i.e., the perceived probability of success/failure = 0.50), indicating that motives consist of two closely related expectation components: cognitive and affective. The cognitive expectation component entails an assessment of the probability of success (P_S_) in solving a task and an assessment of the importance or value of the task. The affective expectation component is a type of prescient feeling that occurs when a mastery situation approaches and presumably accompanies the activation of motives through points of reference in situations that signal a challenge. The difference between scores on the motive to achieve success (Ms) and fear of failure (Mf) reveals whether arousal of an individual’s achievement motive is motivated by success or fear of failure. When activated, the motives have three essential functions: choosing, orienting, and energizing behavior (McClelland, 1987). Emotional expectations will impact a person’s development and regulation of plans and strategies (e.g., level of self-regulated learning) and the direction of their goals (e.g., approach and avoidance). Therefore, an expectation is presumably related to the orientation to goals in a specific mastery situation and is influenced by an emotional state (e.g., hope or fear).

Empirical studies indicate a positive association between scores on the success motive (High Ms) and soccer performance, whereas several studies have negatively related scores on the failure motive (high Mf) to soccer performance. For example, Murr et al.’s (2018) systematic review of longitudinal studies on the predictive value of Ms for future soccer performance found effect sizes between 0.27 ≤ *d* ≤ 0.74 and negative effect sizes between 0.21 ≤ *d* ≤ 0.30 of Mf and soccer performance. Moreover, based on Atkinson’s risk-taking model, the achievement motives (Ms and Mf) will influence the incentive of success or failure on a specific task, while the probability of success will affect the activation of motives (Atkinson and Feather, 1966). A probability of success ≤0.50 is assumed to produce the strongest motivation to master a task (approach motivation) for success motivated individuals. On the other hand, for individuals with a high failure motive, a task of moderate difficulty is likely to motivate them to avoid the task (avoidance motivation), while a very easy task could produce relatively high positive motivation (Atkinson, 1957).

### Achievement goals

According to Elliot (1999), achievement goals are commonly recruited to serve the underlying motive-based motivation by strategically guiding it toward concrete aims that address the underlying motives. *Performance goals* are focused on the demonstration of competence relative to others, whereas *mastery goals* are focused on the development of competence through task mastery (Elliot and McGregor, 2001). In the early development phase of this tradition, theorists generally assumed that performance goals were related to negative processes and consequences, such as giving up more easily in the face of resistance (Dweck, 1986) and having lower intrinsic motivation (Nicholls, 1984). Mastery goals were assumed to be related to several positive processes and consequences, such as great endurance in the face of resistance (Dweck and Leggett, 1988), seeking optimal challenges (Dweck, 1986), and intrinsic motivation (Elliot and Harackiewicz, 1996).

An experiment conducted by Elliot and Harackiewicz (1996) showed that it might be appropriate to divide the performance goal into two goals: first, demonstrating one’s *abilities compared to others’ abilities*, and second, to *avoiding failure or appearing in a negative light*. Studies have since strengthened the assumption that it is appropriate to apply performance-avoidance goals (Elliot, 2006). The trichomous achievement goal model comprised a comparable mastery goal to those from the two goal model and two performance goals; one focused on doing well relative to other (performance-approach), and one focused on not doing poorly relative to others (performance-avoidance). Several subsequent studies have indicated that mastery goals also should be differentiated. In addition to the mastery approach, a mastery avoidance goal has been described to avoid failure. It is defined in mastery terms and entails a reduction or stagnation in developing skills and competencies (the 2 × 2 model; Elliot and McGregor, 2001). Later, a 3 × 2 model was introduced, where mastery-based goals were separated in task and self-based goals (Elliot et al., 2011). In this model, task-based and self-based *mastery goals* focus on the absolute requirements to master the task or activity (i.e., the degree to which one has or has not accomplished the activity) and on learning or development (i.e., the degree to which one is or is not improving), respectively, whereas performance goals are designated as *other-based goals* intended to link the designation more closely to the standard used to define competence. The definition of competence (task/self/other) was then crossed with approach and avoidance to achieve the six goals in the model. More recently, Mascret et al. (2015) have extended the 3 × 2 achievement model to the sport domain. The differentiation of whether one is (or is not) accomplishing the task *per se* (task-based mastery) or on which one is or is not improving on a task/activity assumed to be important in a soccer context.

Most research on achievement goals in soccer have applied a dichotomous model to estimate how mastery and performance orientation relate to behavior and performance. Silva et al. (2010) found regional team players to be more oriented toward performance goals than local players, with no significant differences in mastery goals. Other studies have shown that elite team players score significantly higher on mastery goals than non-elite team players, with no differences in scores for performance goals (Kavussanu et al., 2011). Some studies have shown lack of correlation between mastery goals and future performance (Figueiredo et al., 2009; Huijgen et al., 2014). Meanwhile, Höner and Feichtinger (2016) found a significant positive correlation between mastery goals and performance level, in a 4-year follow-up in young soccer players. Van-Yperen and Duda (1999) found that improvements in performance during a soccer season corresponded to mastery goals. Few studies have investigated the distinction between approach and avoidance goals within a soccer context. Nonetheless, studies in sports suggest that mastery and performance-approach goals are positively related to performance, while no significant relationship has been found between avoidance goals and performance (Van Yperen et al., 2014). However, for athletes under the age of 18 years, the results of a meta-analysis showed that the correlation between performance-approach goals and performance was not significant (Lochbaum and Gottardy, 2015).

### Self-regulated learning

Self-regulated learning (SRL) refers to the process whereby learners systematically orchestrate their thoughts, feelings, and actions to achieve their learning goals (Pintrich, 2000). In SRL, the active role of learners in their own learning process is emphasized. Reflecting on one’s own knowledge and cognitive processes and using this information to regulate and control one’s learning and problem-solving are central aspects. This aspect is referred to as metacognition or thinking about one’s own thinking (Lai, 2011). Zimmerman’s (2006) self-regulated learning model has been operationalized and studied in various contexts. The model consists of three cyclical phases: forethought, performance, and self-reflection. In the forethought phase, learners set their goals, analyze the task requirements, and develop a strategic plan to accomplish those goals. They also assess their self-efficacy and their motivation levels. In the performance phase, learners put their strategic plan into action. They utilize various cognitive and behavioral strategies to complete the task. Feedback and reflection play a crucial role during this phase. In the self-reflection phase, learners evaluate their performance and compare it with their goals. They identify areas of improvement and develop strategies to enhance their future performances. To achieve a world-class level in soccer, it requires not only extensive training, talent, high motivation, and high-quality instructions but also the ability to systematically evaluate and reflect on one’s training practices, level of effort, approach and avoidance motivation, and how the surrounding environment influences one’s learning and performance processes (e.g., McCardle et al., 2018; Young et al., 2023; Zimmerman and Kitsantas, 2005). The extent to which players take responsibility for their own learning in their daily practice is therefore assumed to be of great significance for achieving this type of goals.

Six sub-processes of this phases have been studied in soccer (Toering et al., 2012a) or sports in general (McCardle et al., 2018). Four of these processes are metacognitive: (1) strategic planning and preparations before sessions involves setting goals for a game or practice session, analyzing the opponent’s strengths and weaknesses, and developing plans accordingly; (2) self-monitoring involves observing performance to track progress and stagnation during sessions; (3) evaluation involves comparison of performance and learning outcomes with specific standards; (4) reflection involves looking back on progress over multiple sessions and identifying strengths and weaknesses to gain insight for future learning and devising a plan to improve their skills. Two processes are motivational; (5) regulation of efforts and concentration related to endurance and propensity for mental and physical exertion during sessions, and (6) self-efficacy, the expectancy that they are able to successfully complete a task. Toering et al. (2009) compared soccer players between the ages of 11 and 17 years at an elite level in the Netherlands with regional level players. They found that players with high reflection scores were 4.9 times more likely to play for the top clubs’ academy teams than players with low scores. Moreover, players with high scores on regulation of effort were seven times more likely to play in an academy team than those with low scores. Toering et al. (2012b) investigated self-regulated learning between “international players” and “national players” aged 12–17 years and found no significant differences between the groups for the reported amount of training, albeit regarding reflection. International players scored higher on reflection than national-level players did.

By nature, that type of learning processes depends on volitional processes. Therefore, it is assumed that motivation and direction are required to initiate them (Rheinberg et al., 2000; Zimmerman, 2001). Achievement motives and goals give energy and direction to practice, but how is motivation guided during practice? How a soccer player regulates his thoughts, emotions, and effort during practice will do him/her able to provide higher quality in the practice/training situation.

### Achievement motives, probability of success, achievement goals, and self-regulated learning

Studies show that motives can predict which goals we are oriented toward (Elliot et al., 2011). Moreover, setting mastery-approach goals and performance-avoidance goals are linked to their underlying motives, while setting performance-approach goals may be linked to both the approach motive (Ms) and the avoidance motive (Mf) (Elliot et al., 2010). Setting mastery avoidance goals is associated with high motive to avoid failure (Conroy and Elliot, 2004). Some studies suggest that people with high Ms scores tend to ignore information about how they perform compared to others (performance/other-based goals). Instead, they focus on mastery (task-based approach goals) and their skills development (Self-based approach goals). This may indicate that those with high scores on the success motive tend to set mastery-approach goals for themselves. However, when the mastery goal is divided into task-based and self-based elements, only the task approach appears to be related to the motive to achieve success in a sample of undergraduate students (Diseth, 2015).

Individuals with a high motive to achieve success will activate higher approach motivation and to a greater extent seek out and thrive in challenging situations (probability of success ≤50%). They exhibited a more adaptive mastery pattern than athletes with low scores. Zimmerman (2006) claimed that motivation variables (e.g., high perceived probability of success, activated approach motive, and approach goals) are essential for all self-regulated learning processes. Bandura (1997) suggests that the perceived probability of success is crucial in determining which activities we engage in and the amount of effort and learning processes invested in the activity. Empirical evidence suggests that a high expectancy for success is crucial for athletes’ efforts and endurance in facing difficulties and challenges. For example, studies on endurance sports show that individuals with high success expectancy are more likely to respond with increased effort and fewer negative emotions when they experience competitions going awry than individuals with low (McCormick et al., 2019). Regarding reflection and evaluation, research suggests that athletes with a high success-expectations have a more appropriate attribution pattern for weak performance than do athletes with low (Feltz et al., 2008), that is, it presumably contributes positively to the approach to present and future goals and indirectly to self-regulated learning processes. Moreover, a consistent finding in Van Yperen and Renkema’s three empirical studies from 2008 was that high-performance expectancy is associated with adoption of other-based approach goals, that is, when soccer players consider their skills to be good, it can create a desire to test out how good they are compared to others.

According to Atkinson, in challenging situations (i.e., a task of moderately difficulty, *p*s = 0.50), motives are activated to their maximum. Therefore, it is expected that the influence of motives on motivation is moderated by the perceived likelihood of success or failure. One would thus assume that the interaction between motive and probability of success will influence the strength and direction of goals and possibly also the degree of self-regulated learning. Concerning achievement goals, the theoretical basis suggests that approach goals and mastery (task- and self-based) are related to adaptive learning processes. This is supported by a meta-analysis that examined the relationship between three types of achievement goals: mastery, performance-approach, and performance-avoidance, and self-regulated learning processes: monitoring, evaluation, and self-reaction, across a range of contexts (Cellar et al., 2011). The results showed that mastery-approach goals were positively related to all learning processes, while performance goals (approach and avoidance) were not significantly related. Evidently, the achievement motives indirectly affect learning processes through goals. Among a sample of undergraduate student, Ms. was significantly related to metacognitive self-regulation and mastery-approach goals partially mediated this relationship (Bartels and Magun-Jackson, 2009). The relationship between the fear of failure motive/avoidance goals and self-regulated learning is more uncertain since it is possible that self-regulated learning is also motivated by fear of failure or avoidance goals. For example, performance-avoidance goals were positively related to metacognitive self-regulation (*r* = 0.31, *p* < 0.01) in Bartels and Magun-Jackson (2009) and task-based mastery avoidance goals were positively related to adaptive strategies in Madjar et al.’s (2011) study.

### The present study

Studies on the topic of motivation in soccer have some limitations. First, the dichotomous perspective on achievement goals, whereby a distinction is made between mastery and performance, is dominant (Murr et al., 2018). This study seeks to expand the research on the impact of approach and avoidance goals. Second, there are few empirical studies on the outcomes of achievement motives among talented young footballers who are eligible for national teams. Third, it is uncertain how the probability of success affects the relationships between motives, achievement goals, and self-regulation among young elite soccer players. As far as we know, there are no studies that have examined the relationship between the 3 × 2 goal structure and effective learning processes. Whether the goals predict differently when distinguishing between task and self-mastery is therefore an open question. According to Brunstein and Heckhausen (2008, p. 181), the achievement motivation researchers have, for decades, been more focused on performance and neglected to clarify the connection between motivation and learning. Therefore, we also investigated whether the observed relationship between the antecedents in the hierarchical model and self-regulated learning and performance is transferable to this population. Third, samples in most soccer studies have comprised boys. This study sought to expand the research to include girls and investigate differences in motivation between boys and girls in this population. The study partly comprises a continuation and expansion of a hierarchical motive-goal-behavior model (e.g., Elliot, 1999). Here, it is suggested that an aroused motive (i.e., affective expectation) evokes either approach or avoidance goals, which in turn regulate specific behavior. We therefore seek to answer the question, *“How are achievement motives, the probability of success, and achievement goals related to self-regulated learning and coach-reported performance among the greatest young football talents in Norway?”*

**Figure 1:**
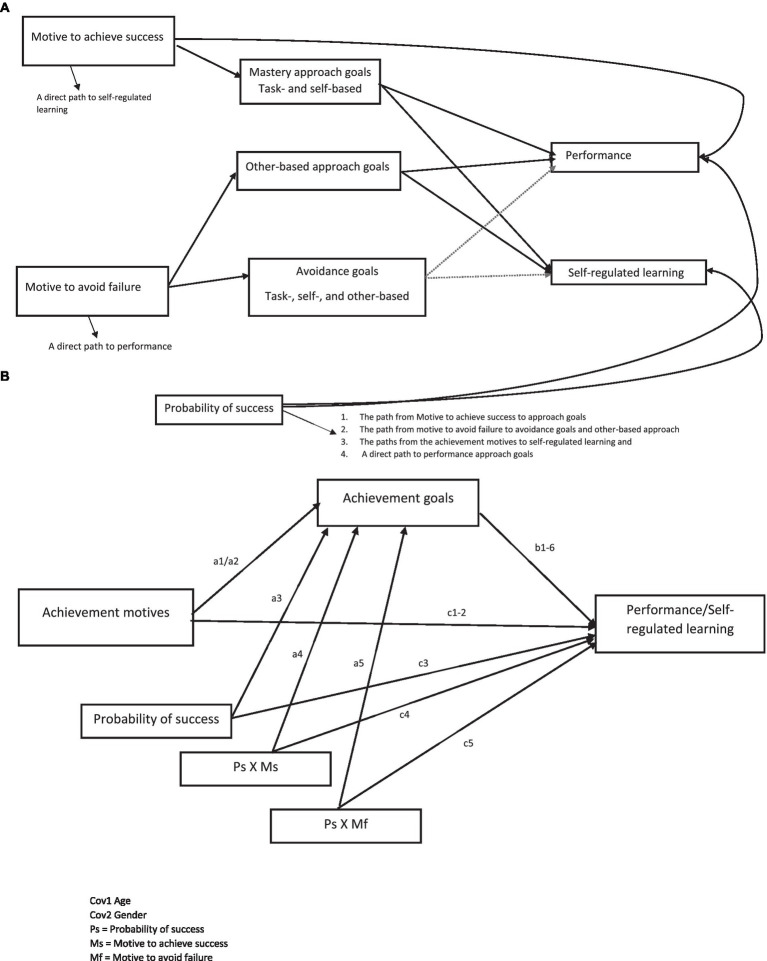
**(A)** The proposed conditional process model. **(B)** The statistical model.

In that context, we hypothesize two effects as part of a conditional process model of motives, achievement goals, and soccer-related outcomes. The first is that the direct effect of achievement motives on soccer-related outcomes (achievement goals, regulation of effort, coach-reported performance, and metacognition) is moderated by the perceived probability of success. The second is that the effect of achievement motives on soccer-related outcomes is mediated by achievement goals. The proposed conditional process model is presented in Figures 1A,B.

Based on this, the following hypotheses have been proposed:

Direct effects:

*H1*: High probability of success positively predicts other-based approach goals, self-regulated learning, and performance (a3, c3).

*H2*: Motive to achieve success positively predicts mastery-approach goals (task and self), performance, and self-regulated learning (a1, c1).

*H3*: Approach goals (task, self, and others) positively predict performance (b1, b2, b3).

*H4*: Mastery-approach goals (task- and self-based) positively predict self-regulated learning (b1, b2).

*H5*: Motive to avoid failure positively relates to avoidance and other-based approach goals (a2).

*H6*: Motive to avoid failure is negatively related to performance (c2).

Moderated effects:

*H7*: Low and medium probability of success intensify the effect of the achievement motives on achievement goals/self-regulated learning (a4, a5, c4, c5).

Mediation/moderated mediation effects:

*H8*: Mastery-approach goals (task and self) mediate the relationship between motive to achieve success on the one hand and performance/self-regulated learning on the other.

*H9*: The mediation effect of achievement goals on the relationship between the motive to achieve success and self-regulated learning is moderated by the probability of success.

Studies of sex and age differences in motivation and learning in youth elite footballer are few (Wachsmuth et al., 2023). Thus, we did not forward specific hypotheses for sex or age differences.

## Methods

### Data collection procedure

Participants comprised the most promising young soccer talents in Norway. The Norwegian Football Association (NFF) holds a national team school talent camp three times per year. The players selected for the camps are considered highly suitable for national youth teams. The selection of players for camps occurs systematically by dividing Norway into five regions with associated constituencies. Constituency officers for player development meet in their regions to nominate players from the region for the talent camp based on their observations during matches and training sessions. After the nominations, cohort managers from the football federation and national team coaches make the final selection based on the nominations and matches they have observed on video and/or live. In 2020, due to COVID-19, only one talent camp was organized for each age group. A total of 257 players were selected for the camps and were divided into 11 teams. There were two teams for boys in the age groups 14, 15, and 16. For girls, there were also two teams for the 14- and 15-year-olds, but only one team for the 16-year-old girls (28 players). Prior to the talent camps, two informational meetings were held: one for the parents of the boys and one for the girls selected for the camps. During these meetings, the research project was briefly presented to all the parents and coaches. In accordance with the guidelines from the Norwegian Centre for Research Data (NSD, registration number 753125/2020), parents of players under 15 years old received an electronic information letter and consent form for participation in the project. After parental consent was granted, players received information letters and had the opportunity to provide consent. All players received a consent form, and those who provided consent automatically received a link to an electronic questionnaire via email or SMS. Time was set aside during the beginning of the camp for the six groups of players to fill out the form, and a data collector was present to provide instructions and answer any questions.

### Participants

Data from 192 players (of the 257 selected, see Table 1) were collected during August 2020, when the Norwegian national team school talent camps was held. There were three age groups with a mean age of 14.9 years (SD = 0.8; range = 14–16). Girls accounted for 42% of the respondents. The response rates for girls and boys selected for the talent camps were 74 and 75%, respectively. Three of the players did not participate due to not receiving permission from their club to attend the camp, for 53 we were unable to reach the parents or youth in front of the camp, and nine consented to participate but were not present when we conducted data collection (due to injuries or illness).

**Table 1:** Descriptive data of the participants.

	Total sample		U16		U15		U14	
	*n* = 192		*n* = 53		*n* = 66		*n* = 73	
	*n*	%	*n*	%	*n*	%	*n*	%
Gender								
Male	110	57.3%	37	69.8%	35	53%	38	52.1%
Female	82	42.7%	16	30.2%	31	47%	35	47.9%

Data were stored with Services for Sensitive Data (TSD) at the University of Oslo. No *a priori* power analysis was conducted for the study. The sample size was determined by number of participants selected for the national team camps for boys and girls in 3-year classes in 2020.

#### Analyses

AMOS version 26 was used for confirmatory factor analysis (CFA). There are various robust methods for analyzing the factor structure. The most appropriate available method in AMOS for non-normally distributed data with a sample size of less than 200 is the maximum likelihood with the Bollen-Stine procedure. For all CFA, we obtained *χ*^2^ statistic based on 5,000 bootstrap samples and applied the procedure suggested by Walker and Smith (2017) to compute model fit indices adjusted in accordance with the Bollen-Stine procedure. While factors were allowed to correlate, we did not allow for correlated errors. Descriptive statistics were calculated for all measures using SPSS version 29. Welch *t*-tests examined sex differences in achievement motives, achievement goals, perceived probability of success, self-regulated learning, and coach-reported performance (see Table 2) since it performs better than Student’s *t*-test whenever sample sizes and variances are unequal between groups and gives the same result when sample sizes and variances are equal (Delacre et al., 2017). Investigating age group differences, we used a one-way ANOVA (see Table 3). The correlations between the study variables were conducted (see Table 4). As a criterion for checking whether our data satisfied a normal distribution, we used the guidelines from Byrne (2016) and Hair et al. (2010), respectively, which state that variables’ skewness and kurtosis scores should fall within a range of ±2 and ± 7 for skewness and kurtosis.

**Table 2:** Sex differences in achievement motives, probability of success, achievement goals, self-regulated learning, and coach reported performance.

				Total		Boys		Girls		Welch *t*-test		
	*N*	Number of item	Response scale	M	SD	M	SD	M	SD	*t*	Df2	Sig.
1. Motive to achieve success	192	5	1–4	18.44	1.59	18.38	1.60	18.51	1.57	0.32	176.76	0.574
2. Motive to avoid failure	192	5	1–4	9.98	3.00	9.46	3.06	10.68	2.78	**8.28**	**182.77**	**0.004**
3. Probability of success	192	7	1–8	34.88	7.76	34.77	7.84	35.02	7.69	0.049	176.40	0.824
4. Task-based approach	192	3	1–7	20.13	1.53	20.11	1.41	20.14	1.69	0.026	155.50	0.872
5. Task-based avoidance	192	3	1–7	15.90	4.70	15.96	4.65	15.80	4.80	0.053	171.62	0.872
6. Self-based approach	192	3	1–7	19.14	2.09	18.90	2.24	19.46	1.82	3.68	188.64	0.057
7. Self-based avoidance	192	3	1–7	15.77	4.74	15.78	4.68	15.75	4.85	0.001	171.62	0.970
8. Other-based approach	192	3	1–7	16.60	4.09	17.09	3.87	15.96	4.30	3.51	163.92	0.058
9. Other-based avoidance	192	3	1–7	14.10	5.18	14.61	4.85	13.40	5.54	2.51	160.83	0.108
10. Planning	192	4	1–5	15.59	2.75	15.88	2.74	15.20	2.73	2.86	174.69	0.093
11. Reflection/evaluation	192	4	1–5	13.31	1.59	13.44	1.49	13.12	1.70	1.89	161.65	0.163
12. Effort	192	3	1–5	13.82	1.25	13.80	1.25	13.85	1.27	0.085	174.69	0.770
13. Coach reported performance	189	1	1–5	3.52	0.94	3.46	0.91	3.60	0.97	1.02	169.28	0.315

Bold values = *p* < 0.05.

**Table 3:** Age differences in achievement motives, probability of success, achievement goals, self-regulated learning, and coach reported performance.

				Total		U15		U16		U17		Welch *F*-test		
	*N*	Number of item	Response scale	M	SD	M	SD	M	ST	M	ST	Welch *F*-test	Df	Sig.
1. Motive to achieve success	192	5	1–4	18.44	1.59	18.59	1.57	18.38	1.64	18.30	1.55	0.58	2, 121.35	0.561
2. Motive to avoid failure	192	5	1–4	9.98	3.00	9.16	2.62	10.79	2.89	10.11	3.36	**6.05**	**2, 115.85**	**0.003**
3. Probability of success	192	7	1–8	34.88	7.76	36.33	7.16	34.14	7.97	33.81	8.11	2.20	2, 118,53	0.115
4. Task-based approach	192	3	1–7	20.13	1.53	20.18	1.33	20.03	1.80	20.17	1.46	0.16	2, 118.11	0.850
5. Task-based avoidance	192	3	1–7	15.90	4.70	16.34	4.63	15.56	4.98	15.70	4.50	0.54	2, 121.65	0.586
6. Self-based approach	192	3	1–7	19.14	2.09	19.23	1.74	19.40	1.82	18.68	2.72	1.40	2, 111.78	0.251
7. Self-based avoidance	192	3	1–7	15.77	4.74	15.78	4.94	16.02	4.73	15.45	4.54	0.22	2, 122,45	0.805
8. Other-based approach	192	3	1–7	16.60	4.09	16.16	4.37	16.62	4.36	17.21	3.24	1.20	2, 125.29	0.304
9. Other-based avoidance	192	3	1–7	14.10	5.18	13.66	5.11	14.50	5.67	14.20	4.65	0.45	2, 122,75	0.640
10. Planning	192	4	1–5	15.59	2.75	15.10	2.96	15.94	2.72	15.85	2.41	1.82	2, 124.66	0.166
11. Reflection/evaluation	192	4	1–5	13.31	1.59	13.05	1.63	13.47	1.53	13.45	1.58	1.46	2, 121.36	0.237
12. Effort	192	3	1–5	13.82	1.25	13.63	1.33	13.91	1.32	13.98	1.03	1.49	2, 124.98	0.230
13. Coach reported performance	189	1	1–5	3.52	0.94	3.41	0.98	3.56	0.96	3.61	0.84	0.84	2, 121.75	0.434

Bold values = *p* < 0.05.

**Table 4:** Descriptive statistics and Correlation between the variables (Pearson’s r).

	*α*	1	2	3	4	5	6	7	8	9	10	11	12	13
1. Motive to achieve success	0.76	1	−0.18^**^	0.10	0.29^**^	−0.01	0.25^**^	−0.00	0.07	−0.02	0.31^**^	0.33^**^	0.31^**^	−0.07
2. Motive to avoid failure	0.69		1	−0.22^**^	−0.06	0.20^**^	0.08	0.27^**^	0.15^*^	0.28^**^	−0.07	−0.04	−0.15^*^	0.05
3. Probability of success	0.82			1	0.10	0.01	−0.02	−0.06	0.11	0.00	0.27^**^	0.18*	0.22^**^	0.26^**^
4. Task-based approach	0.72				1	0.22^**^	0.51^**^	0.20^**^	0.36^**^	0.25^**^	0.21^**^	0.25^**^	0.34^**^	0.08
5. Task-based avoidance	0.81					1	0.33^**^	0.79^**^	0.36^**^	0.70^**^	0.12	0.11	−0.01	−0.05
6. Self-based approach	0.67						1	0.32^**^	0.35^**^	0.31^**^	0.16^*^	0.23^**^	0.30^**^	0.07
7. Self-based avoidance	0.83							1	0.27^**^	0.72^**^	0.12	0.16^*^	−0.00	−0.04
8. Other-based approach	0.86								1	0.63^**^	0.19^**^	0.16^*^	0.16^*^	0.09
9. Other-based avoidance	0.90									1	0.12	0.13	−0.04	0.00
10. Planning	0.82										1	0.55^**^	0.46^**^	0.00
11. Reflection/evaluation	0.68											1	0.40^**^	0.07
12. Effort	0.68												1	0.06
13. Coach reported performance	–													1

*α* = Cronbach alpha; *p < 0.05, **p < 0.01.

We used PROCESS 4.0 Macro for SPSS to conduct moderation analysis testing probability of success as a moderator of the relationships of the motives with achievement goals, self-regulated learning, and coach-reported performance during the talent camp (Model 2; Hayes, 2022). To facilitate interpretability, a final set of analyses was included if only one of the interaction terms was significant (Model 1). Statistically significant interactions were probed by estimating effects at high (84th percentile), moderate (50th percentile), and low (16th percentile) levels of the achievement motives and by using the Johnson-Neyman technique to graph conditional effects. Second, a moderation mediation process model was examined (Model 10; Hayes, 2022) to estimate the direct and indirect effects of achievement motives on dimensions of self-regulated learning and performance with probability of success moderating these effects. For all models, we conducted four separate analyses—for self-regulation of effort, planning, reflection/evaluation, and for coach-rated performance. In all models, achievement motives were a focal predictor, achievement goals were treated as a mediator, and one of the dimensions of self-regulated learning and coach-related performance was the outcome variable. We included gender and age group as covariate in the final model. Third, if moderated mediation was not significant, we used a simple mediation model (Model 4) to test whether there was evidence of an indirect effect when no moderator was included in the analysis. In the last stripped model, only those mediators that showed a significant correlation with the outcome variable were included. In all models, bootstrap procedure with 5,000 iterations was used, and the significant confidence interval was 95%. A bootstrapped 95% CI that did not contain zero indicated a significant indirect effect. To interpret the magnitude of the effect sizes, we use the guidelines for personality research by Funder and Ozer (2019), with *r* = 0.05, *r* = 0.10, *r* = 0.20, *r* = 0.30, and *r* = 0.40 or greater corresponding to very small, small, medium, large, and very large effects, respectively.

### Instruments

#### Achievement motives

To measure achievement motives, we used the Achievement Motives Scale-Sport, a revised sport-specific 10-item edition (AMS-S; Elbe and Wenhold, 2005; Wenhold et al., 2009), of the original 30 items AMS (Gjesme and Nygård, 1970). The instrument measures the strength of the motive to achieve success (Ms) and avoid failure (Mf). As in Höner and Feichtinger (2016), the respondents rated how well each statement matched their usual reactions in soccer-specific situations, ranging from 1 (“completely disagree”) to 4 (“completely agree”). To interpret the AMS scores, Elbe and Wenhold (2005) calculate the difference of the two scores, the net hope = Ms – Mf. A positive net hope indicates that the soccer players enjoy challenging situations during matches or training situations, whereas a negative value indicates that the players find such situations unpleasant. In this sample, the mean net hope = 8.45, and only two out of 192 had a negative value. Satisfactory internal reliability was observed for Ms (*α* = 0.76) and Mf (*α* = 0.69). A confirmatory factor analysis (CFA) produced the expected two factors of motive for success and motive to avoid failure [Bollen-Stine *χ*^2^ (df = 31) = 29.58 (*p* = 0.20), Bs scaling factor = 1.427, CFI = 0.988, IFI = 0.987, TLI = 0.995, RMSEA = 0.035].

#### Probability of success

Various methods have been used to measure probability of success (Ps) in achievement motivation research (Brunstein and Heckhausen, 2008). In our case, where there is competition among 50 other players to secure a place in a national team squad, it is most relevant to use a social comparison standard where the Ps is largely dependent on how a player rates his or her ability relatively to that of others. To contextualize the subjective probability of success, we wanted a measure where the players assessed themselves on a set of soccer skills that experts in the field assume to be central to achieving success and rank them compared against the top 50 players in their age group in Norway skills. Items from Van Yperen’s performance level measure were chosen (VanYperen, 1995; Van-Yperen and Duda, 1999). The soccer skills in measure were developed by Ajax technical staff and assess players’ abilities in important areas for soccer performance. In this study, we adapted and reworked the introduction and response options to suit our purpose. The players were asked to assess their soccer skills compared to the top 50 players in Norway in their age group (corresponding number of players as in the different age groups at the national team training camp), and the item responses were as follows: (1) not among the top 50, (2) among the 50–41, (3) 40–31, (4) 30–21, (5) 20–11, (6) among the top 10, (7) among the top 5, (8) the best. The measure includes seven items: six detailed (speed; endurance/conditioning; strength, technique, tactical ability; understanding of the game, and mentality) (how mentally strong on the football field), and one global appraisal (an overall appraisal of own talent) of soccer skills. Satisfactory internal reliability was observed (*α* = 0.82). A CFA produced the expected probability of success factor with Bollen-Stine *χ*^2^ (df = 14) = 23.685 (*p* = 0.00), Bs scaling factor = 3.696, CFI = 0.980, IFI = 0.980, TLI = 0.970, and RMSEA = 0.06.

#### Achievement goals

We used the soccer version of the 3 × 2 Achievement Goals Questionnaire (AGQ, Elliot et al., 2011). Mascret et al. (2015) translated and validated the AGQ in the context of a specific sport. The AGQ evaluates six types of goals, each consisting of three indicators. In this study, we use a translated and validated Norwegian version of the AGQ (Diseth, 2015). The instruction in this study is, however, aimed at the types of goals you have or may not have when you play soccer. Participants rated the extent to which they agreed or disagreed that the different goals were suitable for them regarding playing soccer, ranging from 1 (“strongly disagree”) to 7 (“strongly agree”). Satisfactory to high levels of internal reliability were observed for the achievement goals (0.67–0.90, see Table 4). Consistent with the 3 × 2 model, the six-factor model showed satisfactory fit to our data (Bollen-Stine *χ*^2^ (df = 120) = 156.053, *p* = 0.015, Bs scaling factor = 1.673, CFI = 0.982, IFI = 0.982, TLI = 0.977, RMSEA = 0.06).

#### Soccer-specific self-regulated learning

We created a custom soccer-specific measure of self-regulated learning based on items from two established questionnaires (McCardle et al., 2018; Toering et al., 2013). The extended SRL-SP measures four sub-processes related to self-regulated learning: planning, reflection/evaluation, effort, and self-efficacy. The relationship between self-efficacy, motivation, and goals has already been examined in several studies (see e.g., Bjørnebekk et al., 2013). To keep questionnaires short and informative, we only used the scales to measure planning, evaluation/reflection, and regulation of effort. The items were reformulated from sports to a soccer context consisting of four items for planning, four for reflection/evaluation, and three for effort. The effort and planning items were chosen based on how high they loaded on the factor in McCardle et al.’s measure models (2018). Evaluation/reflection items were selected based on their high loadings in the extended model and their satisfactory performance in Toering et al.’s football study (2013) as this was the factor that characterized top players. For all three, they also covered the breadth of the concepts that were measured (for items and factor loadings, see Appendix Table 1). Participants rated how well the various statements suited them, ranging from 1 (“does not fit at all”) to 5 (“fits very well”). The results showed that the specified model fit the data well [Bollen-Stine *χ*^2^ (df = 41) = 29.582 (*p* = 0.06), Bs scaling factor = 1.427, CFI = 0.988, IFI = 0.987, TLI = 0.995, RMSEA = 0.035]. Satisfactory internal reliability was observed for planning (*α* = 0.82), evaluation/reflection (*α* = 0.68), and effort (*α* = 0.68).

#### Coach-reported performance

At the talent camp, all participants were classified based on their performance during their stay. The classification is well incorporated into the national school team and undertaken over a long period. Constituency managers for player development and national team coaches operate as coaches during their stays. There are two to four coaches for each player group, comprising 20–25 players. During the talent camp, which lasted 5 days, all groups played two matches and participated in two to three training sessions. After the talent camp, coaches in each group classified their players based on the following scale: 1—“highly relevant for a national team eleven,” 2—“highly relevant for a national team squad,” 3—“highly relevant for a shadow national team eleven,” 4—“highly relevant for a shadow national team squad,” and 5—“worth following.”

## Results

Regarding the motive to achieve success, the task- and self-based approaches, skewness, and kurtosis suggest that the variables deviated from a normal distribution. The motive to achieve success (skewness = −2.92, Kurtosis = 13.02) and task-based approach (skewness = −2.84, Kurtosis = 11.88) had skewness and kurtosis over the recommended rule of thumb. The other variables were within the acceptable range. Both mirrored logarithmic and inverse transformations were attempted to improve the distribution as it approached normal. After the transformation, skewness and kurtosis were re-examined, and the correlation between the transformed and original scales was considered. The results showed that the mirrored inverse transformation 1/ (K – x) produced the most satisfactory results (For MS skewness = −0.52 and kurtosis = −0.25/task-based performance skewness = −1.03 and kurtosis = −0.26). This transformation was performed and used in the moderation and mediation analysis. A variance inflation factor (VIF) analysis was performed to check for multicollinearity. This analysis showed that the VIF ranged from 1.17 and 3.90, which is below the recommended limit (VIF > 5, Chatterjee and Simonoff, 2013).

### Sex and age differences, descriptive statistics, and correlations between variables

First, Welch *t*-tests were presented to illustrate the overall relationships and investigate whether there were differences between boys and girls. F-tests were presented for differences for age groups.

The independent sample *t*-tests found one significant sex difference (see Table 2): girls reported higher scores on the motive to avoid failure (*M* = 2.14, SD = 0.56) than boys (*M* = 1.89, SD = 0.61, *t* (190) = −2.88, *p* < 0.01). The boys had higher score than the girls on other-based goals, respectively, *M* = 14.61 vs. *M* = 13.40, for avoidance and, *M* = 17.09 vs. *M* = 15.96, for approach, and in terms of planning training and match situations in advance (*M* = 15.88 vs. *M* = 15.20), while the girls scored higher on self-based approach goals (*M* = 18.90 vs. *M* = 19.46). However, these latter differences were only marginally significant. In addition, the youngest players scored lower on the motive to avoid failure than older players did (see Table 3). The analyses showed no significant mean differences in scores by sex or age group regarding the other variables.

In sum, among talented footballers, the findings suggest that girls’ scores on the motive to avoid failure were higher than those of boys. However, girls’ and boys’ scores were relatively similar for football-specific self-regulated learning, perceived probability of success, achievement goals, and the motive to achieve success.

The results, presented in Table 4, showed several significant correlations between the predictors and mediators that supported our assumptions; The motive to achieve success was moderately positively correlated with task- and self-based approach goals (*r* = 0.29 and 0.25) but not significantly related to other approach goals. The motive to avoid failure was moderately positively associated with all three avoidance goals (task-based *r* = 0.20, self-based *r* = 0.27, other-based *r* = 0.28) and weakly to other-based approach goals (*r* = 0.15). When it comes to the relationship between the predictors and the outcomes variables, the motive to achieve success was positively related to planning (*r* = 0.31), reflection/evaluation (*r* = 0.33), and regulation of effort (*r* = 0.31) and the relationships were large. The motive to avoid failure, however, was only significantly related to effort, and the association was small (*r* = −0.15). Moreover, all the approach goals were moderately positively associated with self-regulation of planning, effort, and reflection/evaluation. For the avoidance goals, the only significant relationship was between self-based avoidance goals and reflection/evaluation. However, except for the association to probability to success (*r* = 0.26), non-significant correlations were observed between coach-reported performance and of the predictors or mediators.

### The achievement goals

The moderation mediation analyses (Model 10) showed that in the first part of the model (i.e., the paths from the predictors to the mediators, see Table 5a), the motive to achieve success was positively related to task-based (*b* = 0.288, SE = 0.07, *p* = 0.000, 95% CI [0.148–0.428]) and self-based approach goals (*b* = 0.241, SE = 0.07, *p* < 0.001, 95% CI [0.098–0.384]). However, the association between the motive to achieve success and other-based approach goals was not significant (*b* = 0.106, SE = 0.07, *p* = 0.14, 95% CI [−0.036–0.250]). Similarly, the motive to avoid failure was significantly related to the avoidance goals: task-based (*b* = 0.24, SE = 0.08, *p* < 0.002, 95% CI [0.089–0.392]), self-based (*b* = 0.296, SE = 0.08, *p* < 0.000, 95% CI [0.148–0.445]), and other-based avoidance goals (*b* = 0.338, SE = 0.07, *p* < 0.000, 95% CI [0.192–0.483]). Conversely to Ms, Mf was positively related to other-based approach goals (*b* = 0.223, SE = 0.08, *p* < 0.004, 95% CI [0.075–0.372]). The probability for success was only significantly related to other-based approach goals (*b* = 0.184, SE = 0.07, *p* = 0.013, 95% CI [0.040–0.327]).

**Table 5a:** Path coefficients, standard errors, and confidence intervals – the mediators (Hayes Model 10).

	Task-based approach goal			Self-based approach goal			Other-based approach goal			Task-based avoidance goal			Self-based avoidance goal			Other-based avoidance goal		
Predictors	Coeff	SE	95% C. I.	Coeff	SE	95% C. I.	Coeff	SE	95% C. I.	Coeff	SE	95% C. I.	Coeff	SE	95% C. I.	Coeff	SE	95% C. I.
a1 Ms	0.288***	0.07	0.148–0.428	0.241***	0.07	0.098 –0.384	0.106	0.07	−0.036 –0.250	0.024	0.07	−0.120 to 0.168	0.062	0.07	−0.079 – 0.203	0.056	0.07	−0.082 – 0.196
a2 Mf	0.002	0.08	−0.146–0.150	0.091	0.08	−0.059 to 0.242	0.223**	0.08	0.075 to 0.372	0.240***	0.08	0.089 to 0.392	0.296***	0.08	0.148 – 0.445	0.338***	0.07	0.192 – 0.483
a3 Ps	0.089	0.07	−0.054 to 0.232	−0.027	0.07	−0.172 to 0.119	0.184*	0.07	0.040 –0.328	0.055	0.07	−0.091 to 0.202	0.017	0.07	−0.127 – 0.160	0.100	0.07	−0.041 – 0.241
a4 Ms x Ps	−0.148*	0.06	−0.269 to −0.028	−0.096	0.06	−0.219 to 0.026	−0.008	0.06	−0.129 to 0.114	−0.131*	0.06	−0.254 to −0.008	−0.109	0.06	−0.230 – 0.012	−0.072	0.06	−0.190 – 0.047
a5 Mf x Ps	−0.094	0.06	−0.219 to 0.032	−0.070	0.06	−0.198 to 0.057	−0.130*	0.06	−0.256 to −0.004	−0.082	0.06	−0.210 to 0.046	−0.146*	0.06	−0.272 – −0.021	−0.155*	0.06	−0.278 – −0.031
Gender	0.089	0.15	−0.198 to 0.375	0.220	0.15	−0.072 to 0.511	−0.347*	0.15	−0.635 to −0.059	−0.146	0.15	−0.439 to 0.147	−0.131	0.15	−0.418 – 0.156	–0.364*	0.14	−0.646 – −0.082
Age	0.009	0.09	−0.167 to 0.185	−0.068	0.09	−0.247 to 0.111	0.087	0.09	−0.090 to 0.264	−0.146	0.09	−0.325 to 0.034	−0.115	0.09	−0.291 – 0.061	−0.034	0.09	−0.207 – 0.139
Constant	−0.060	0.20	−0.448 to 0.338	0.030	0.21	−0.375 to 0.435	−0.044	0.20	−0.445 to 0.357	0.334	0.21	−0.074 to 0.741	0.253	0.20	−0.146 – 0.652	0.194	0.20	−0.198 – 0.686
		*R*^2^ = 0.124			*R*^2^ = 0.091			*R*^2^ = 0.110		*R*^2^ = 0.081			*R*^2^ = 0.118				*R*^2^ = 0.148	
	*F* (7, 184) = 3.71, *p* = 0.001			*F* (7, 184) = 2.63, *p* = 0.013			*F* (7, 184) = 3.27, *p* = 0.003			*F* (7, 184) = 2.32, *p* = 0.027			*F* (7, 184) = 3.51, *p* = 0.002			*F* (7, 184) = 4.58, *p* = 0.000		

*p < 0.05. **p < 0.01. ***p < 0.001, two-tailed. *N* = 192; SE, standard error; CI, 95% confidence interval. Gender: 0 = Male; 1 = Female. Bold values = *p* < 0.05.

#### Regulation of effort

Concerning regulation of effort, the results showed significant direct effects (c1, see Table 5b) of the motive to achieve success (*b* = 0.233, SE = 0.08, *p* < 0.005, 95% CI [0.066–0.398]) and of the probability of success (*b* = 0.247, SE = 0.08, *p* < 0.005, 95% CI [0.082–0.412]). The test of the highest order unconditional interactions indicated that the interactions between the motives and probability of success were not significant. The mediation process was therefore not significantly dependent of the probability of success. For the individual mediators’ contributions, the results showed the following: a significant effect of adoption of task-based approach (*b* = 0.211, 95% [0.006–0.133]) and self-based approach goals (*b* = 0.297, 95% CI [0.100–0.494]). Approximately 32% of the variance in regulation of effort and concentration was accounted for by the moderated mediation model (*F* (13, 178) = 6.37, *p* = 0.000). In the stripped model, the total indirect effect was significant (ie = 0.131 BootSE 0.05, 0.055–0.233). Two of the three approach goals were found to contribute significantly to the overall indirect effect: task-based approach (*b* = 0.068, BootSE = 0.035; 95% CI [0.011–0.153]) and self-based approach goals (*b* = 0.065, BootSE = 0.033, 95% CI [0.012–0.140]).

**Table 5b:** Path coefficients, standard errors, and confidence intervals – the outcome variables (Hayes Model 10).

		Effort		Evaluation/reflection				Planning		Coach rated performance		
Predictors	Coeff	SE	95% C. I.	Coeff	SE	95% C. I.	Coeff	SE	95% C. I.	Coeff	SE	95% C. I.
c1 Ms	0.233**	0.08	0.066 to 398	0.490***	0.11	0.267 to 0.713	0.809***	0.19	0.429 to 1.189	−0.130	0.07	−0.271 to 0.012
c2 Mf	−0.085	0.09	−0.262 to 0.092	0.054	0.12	−0.184 to 0.292	0.086	0.21	−0.320 to 0.491	0.073	0.08	−0.078 to 0.223
c3 Ps	0.247**	0.08	0.082 to 0.412	0.287*	0.11	0.066 to 0.509	0.737***	0.19	0.360 to 1.114	0.276***	0.07	0.135 to 0.417
c4 Ms x Ps	−0.105	0.07	−0.244 to 0.034	−0.154	0.09	−0.341 to 0.032	−0.103	0.16	−0.421 to 0.214	−0.009	0.06	−0.127 to 0.109
c5 Mf x Ps	−0.035	0.07	−0.179 to 0.110	−0.206*	0.09	−0.400 to −0.012	0.021	0.16	−0.309 to 0.351	−0.024	0.06	−0.146 to 0.099
b1 Task-appr	0.211*	0.10	0.016 to 0.406	0.028	0.13	−0.234 to 0.286	0.078	0.23	−0.368 to 0.524	0.072	0.08	−0.094 to 0.239
b2 Self-appr	0.297**	0.10	0.100 to 0.494	0.189	0.13	−0.076 to 0.453	0.304	0.23	−0.147 to 0.755	0.058	0.09	−0.112 to 0.228
b3 Other-appr	0.143	0.12	−0.089 to 0.374	0.048	0.16	−0.262 to 0.359	0.216	0.27	−0.313 to 0.745	0.056	0.10	−0.147 to 0.259
b4 Task-avoid	−0.106	0.14	−0.377 to 0.165	−0.101	0.18	−0.460 to 0.267	0.033	0.31	−0.586 to 0.652	−0.108	0.12	−0.339 to 0.123
b5 Self-avoid	0.149	0.15	−0.142 to 0.440	0.269	0.20	−0.122 to 0.660	0.382	0.34	−0.284 to 1.048	−0.012	0.12	−0.259 to 0.234
b6 Other-avoid	−0.295	0.15	−0.599 to 0.009	−0.084	0.21	−0.495 to 0.321	−0.290	0.35	−0.985 to 0.406	0.002	0.13	−0.260 to 0.256
Cov1 Gender	−0.000	0.17	−0.335 to 0.335	−0.367	0.23	−0.817 to 0.083	−0.766*	0.39	−1.532 to −0.001	0.139	0.15	−0.148 to 0.426
Cov2 Age	0.261*	0.10	0.061 to 0.462	0.240	0.14	−0.029 to 0.510	0.541*	0.23	0.082 to 1.000	0.123	0.09	−0.048 to 0.294
Constant	13.33***	0.23	12.878 to 13.781	12.98***	0.31	12.373 to 13.586	14.910***	0.52	13.878 to 15.943	3.218***	0.20	2.831 to 3.605
		*R*^2^ = 0.318			*R*^2^ = 0.233			*R*^2^ = 0.258			*R*^2^ = 0.129	
	*F* (13, 178) = 6.37 *p* = 0.000			*F* (13, 178) = 4.15 *p* = 0.000			*F* (13, 178) = 4.77, *p* = 0.000			*F* (13, 175) = 2.00, *p* = 0.023		

*p < 0.05. **p < 0.01. ***p < 0.001, two-tailed. *N* = 192; SE, standard error; CI, 95% confidence interval. Gender: 0 = Male; 1 = Female.

#### Evaluation/reflection

The results showed a significant direct effect of the motive to achieve success (*b* = 0.490, SE = 0.11, *p* = 0.000, 95% CI [0.267–0.713]) and of the probability of success (*b* = 0.287, SE =0.11, *p* = 0.011, 95% CI [0.066–0.509]) concerning use of the metacognitive strategies of reflection and evaluation. However, the moderated mediator contributions were not significant. Approximately 23% of the variance in reflection/evaluation was accounted for by the model [*F*(13, 178) = 4.15, *p* < 0.000]. The indirect effect in the stripped model was also non-significant (ie = 0.081, BootSE 0.05; [95% CI -0.006–0.200]).

#### Planning

The results showed a significant direct effect of the motive to achieve success concerning evaluation (*b* = 0.809, SE = 0.19, *p* = 0.000, 95% CI [0.429–1.189]) and of the probability of success (*b* = 0.737, SE = 0.19, *p* = 0.000, 95% CI [0.360–1.114]). The moderated mediation was not significant.

Approximately 26% of the variance in planning was accounted for by the model [*F*(13, 178) = 4.77, *p* = 0.000]. In the striped model, the total indirect effect was significant (ie = 0.122, Boot SE 0.07, 0.004–0.285). However, none of the individual mediator contributions were significant.

#### Coach-reported performance

The probability of success was the only significant contributions (*b* = 0.276, SE = 0.07, *p* = 0.000, 95% CI [0.135–0.417]). Approximately 13% of the variance in coach-rated performance was accounted for by the model (F 13, 175 = 2.00, *p* = 0.23) (Tables 6, 7).

**Table 6:** Testing the moderating effect of probability of success on the relationship between the achievement motives (Ms and Mf) and the six subtypes of achievement goals.

	Task-based approach goal			Self-based approach goal			Other-based approach goal			Task-based avoidance goal			Self-based avoidance goal			Other-based avoidance goal		
Predictors	Coeff	SE	95% C. I.	Coeff	SE	95% C. I.	Coeff	SE	95% C. I.	Coeff	SE	95% C. I.	Coeff	SE	95% C. I.	Coeff	SE	95% C. I.
a1 Ms	0.290***	0.07	0.152 to 0.430	0.262***	0.07	0.121 to 0.404	0.089	0.07	−0.054 −0. 231	0.023	0.07	−0.120 to 0.167	0.061	0.07	−0.080 to 0.201	0.043	0.07	−0.097 to 0.182
a2 Mf	0.013	0.07	−0.129−0.155	0.127	0.07	−0.017 to 0.271	0.191**	0.07	0.045 to 0.336	0.211**	0.07	0.064 to 0.357	0.271***	0.07	0.128 to 414	0.292***	0.07	0.150 to 434
a3 Ps	0.091	0.07	−0.051 to 0.232	0.040	0.07	−0.104 to 0.183	0.168*	0.07	0.023 to 314	0.064	0.07	−0.082 to 0.210	0.023	0.07	−0.120 to 0.165	0.094	0.07	−0.048 to 0.236
a4 Ms x Ps	−0.147*	0.06	−0.266 to −028	−0.076	0.06	−0.196 to 0.045	−0.022	0.06	−0.144 to 0.100	−122	0.06	−0.244 to 0.001	−0.102	0.06	−0.221 to 0.018	−0.076	0.06	−0.195 to 0.043
a5 Mf x Ps	−0.091	0.06	−0.129 to 0.155	−0.133*	0.06	−0.259 to −0.007	−0.143*	0.06	−0.271 to −0.016	−0.081	0.06	−0.209 to 0.047	−0.146*	0.09	−0.271 to −0.020	−0.164**	0.06	−0.289 to −0.040
Constant	−0.005	0.07	−0.143 to 0.133	−0.022	0.07	−0.162 to 119	−0.029	0.07	−0.171 to 0.113	−0.005	0.07	−0.148 to 0.137	−0.022	0.07	−0.161 to 0.118	−0.028	0.07	−0.167 to 0.110
		*R*^2^ = 0.122			*R*^2^ = 0.096			*R*^2^ = 0.074			*R*^2^ = 0.066			*R*^2^ = 0.108			*R*^2^ = 0.118	
	*F* (5, 186) = 5.17, *p* = 0.000			*F* (5, 185) = 3.95, *p* = 0.002			*F* (5, 186) = 2.99, *p* = 0.013			*F* (5, 186) = 2.62, *p* = 0.026			*F* (5, 186) =4.48, *p* = 0.001			*F* (5, 186) = 5.00, *p* = 0.000		

*p < 0.05; **p < 0.01; ***p < 0.001. *N* = 192; SE, standard error. Sex: 0 = Boys; 1 = Girls.

**Table 7:** Testing the moderating effect of probability of success on the relationship between the achievement motives (Ms and Mf) and three types of soccer-specific self-regulated learning as well as coach rated performance.

Regulation of effort					Planning		Evaluation/reflection			Coach reported performance		
Predictors	Coeff	SE	95% C. I.	Coeff	SE	95% C. I.	Coeff	SE	95% C. I.	Coeff	SE	95% C. I.
a1 Ms	0.367***	0.08	0.196 to 0.537	0.890***	0.19	0.520 to 10.259	0.538***	0.11	0.324 −0.751	−0.090	0.07	−0.224 to 0.044
a2 Mf	−0.067	0.09	−0.241 to 0.107	0.146	0.19	−0.231 to 0.523	0.090	0.11	−0.128 to 0.308	0.088	0.06	−0.048 to 0.224
a3 Ps	0.233***	0.09	0.060 to 0.407	0.697***	0.19	0.321 to 10.073	0.256*	0.11	0.041 to 476	0.276***	0.07	0.140 to 0.413
a4 Ms x Ps	−0.167*	0.07	−0.312 to −021	−0.228	0.16	−0.543 to 0.088	−0.212*	0.09	−0.395 to −0.030	−0.021	0.06	−0.135 to 0.093
a5 Mf x Ps	−0.067	0.08	−0.219 to 0.085	−0.089	0.17	−0.259 to −0.007	−0.264**	0.10	−0.454 to −0.073	−0.031	0.06	−.150 to 0.088
Constant	13.852***	.09	−13.656 to 16.994	15.597***	0.19	15.231 – 15.963	13.271***	0.11	13.059 – 13.483	3.514***	0.07	3.381 – 3.657
		*R*^2^ = 0.162			*R*^2^ = 0.180			*R*^2^ = 0.177			*R*^2^ = 0.089	
	*F* (5, 186) = 7.17, *p* = 0.000			*F* (5, 185) = 8.15, *p* = 0.000			*F* (5, 186) = 8.02, *p* = 0.000			*F* (5, 186) = 3.59, *p* = 0.004		

*p < 0.05. **p < 0.01. ***p < 0.001. *N* = 192; SE = standard error. Sex: 0 = Boys; 1 = Girls.

### Probability of success as a moderator of the achievement motives effect on achievement goals and self-regulated learning

**Figure 2:**
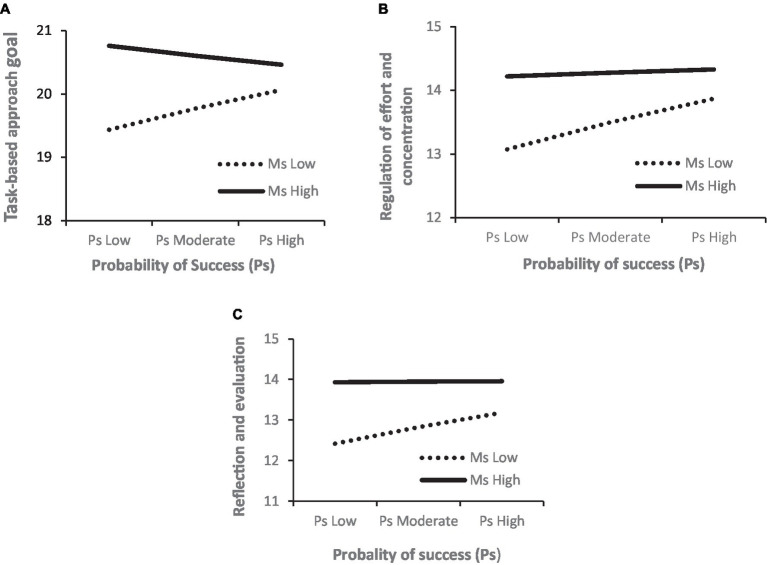
Moderating effect of probability of success on the relationship between the motive to achieve success and **(A)** task-based approach goals, **(B)** effort and concentration, and **(C)** evaluation and reflection.

The moderation analysis revealed that the motive to achieve success effect on task-based approach goals (*b* = −0.147, ∆R^2^ = 0.025, *p* = 0.022), regulation of effort and concentration (*b* = −0.167, ∆R2 = 0.021, *p* = 0.022), and evaluation/reflection (*b* = −0.167, ∆R2 = 0.015, *p* = 0.07) were dependent of the probability of success. The Johnson-Neyman technique indicated that for soccer players with probability of success above 0.77 for task-based approach (21.4% of the players), 1.04 for regulation of effort (16.2% of the players), and 1.23 for evaluation/reflection (7.29%), the probability of success was not related to Ms effect on task-based approach goals (Figure 2A), regulation of effort (Figure 2B), and evaluation/reflection (Figure 2C). In short, it appears that regulation of effort and concentration and use metacognitive strategies to evaluate and reflect during training and competition is only dependent on high probability of success when the motive for success is from moderate to low. Regarding adaption of task-based approach goals, low probability of success seems to increase the tendency for those with a high motive for success but decreases it for those with low.

**Figure 3:**
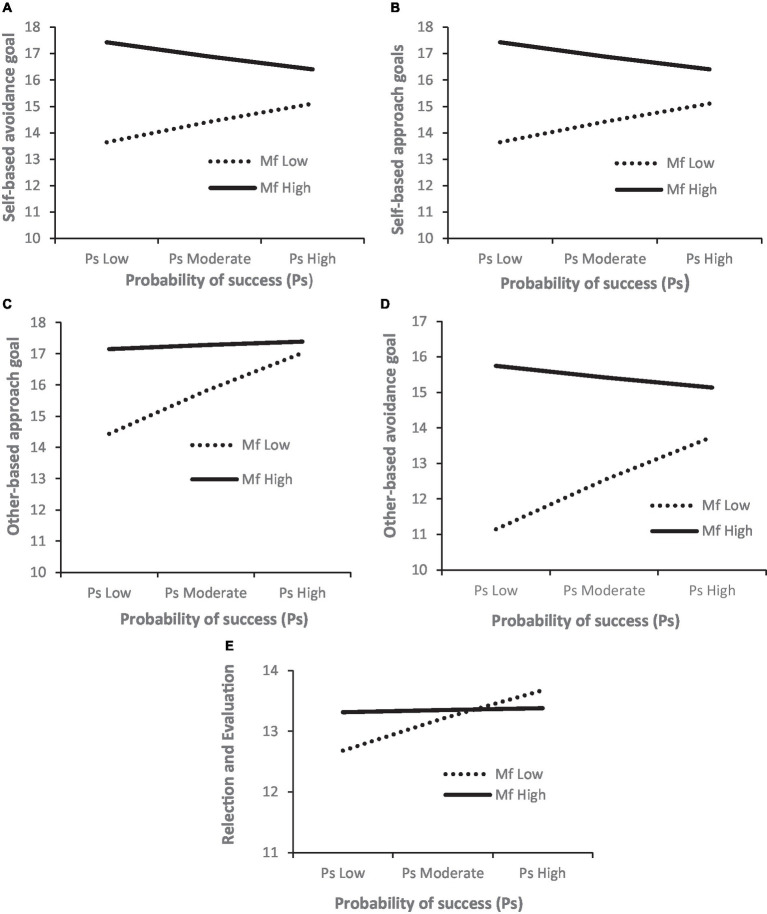
Moderating effect of probability of success on the relationship between the motive to avoid failure and **(A)** self-based avoidance goals, **(B)** self-based approach goals, **(C)** other-based approach goals, **(D)** other-based avoidance goals, and **(E)** evaluation and reflection.

The motive to avoid failure influences adaption of all the achievement goals apart from the task-based approach goal. However, the influence of the fear of failure motive on self- and other-based goals (both approach and avoidance) depends on the probability of success: self-based approach (*b* = −0.133, ∆R^2^ = 0.018, *p* = 0.059) and avoidance goals (*b* = −0.146, ∆R^2^ = 0.020, *p* = 0.044), other-based approach (*b* = −0.143, ∆R^2^ = 0.024, *p* = 0.030) and avoidance goals (*b* = −0.164, ∆R^2^ = 0.028, *p* = 0.017). The difference between high and low Mf is greatest when the probability of success is low for adaption of all four types of achievement goals. Those with low fear of failure increases their adoption of self- and other-based goals when the probability of success is high. However, those with high fear of failure reduce their adoption of self-based goals when the probability of success is high, whereas their adoption of other-based goals remains high despite the probability of success. Furthermore, it appears that when the motive to avoid failure is low, it is only at a high probability of success that regulation of effort and concentration is high (*b* = −0.264 ∆R2 = 0.025, *p* = 0.020). The Johnson-Neyman technique indicated that for soccer players with probability of success above 0.75 for self-based avoidance (21.4% of the players), −0.15 for self-based approach (61.5%), 0.28 for other-based approach (38.5%),0.77 for other-based avoidance (21.4%), and − 0.74 for reflection/evaluation (77.6%), the probability of success was not related to the motive to avoid failures effect on self-based avoidance (Figure 3A), self-based approach (Figure 3B), other-based approach (Figure 3C), other-based avoidance (Figure 3D), and evaluation/reflection (Figure 3E).

## Discussion

In this study, we investigated how achievement motives, probability of success, and achievement goals are related to coach-rated performance and self-regulated learning of 13- to 16-year-old players eligible for the Norwegian national football team. A quantitative methodology with a cross-sectional design was used to investigate these issues. The sample differed from previous studies by including both boys and girls. We therefore also aimed to investigate sex differences in motivation. The *t*-test showed a significant difference between the sexes regarding motive to avoid failure. Girls reported significantly higher scores than boys, while there were no differences between sexes in scores on the motive to achieve success, which supports previous studies on Norwegian youth athletes (Halvari and Thomassen, 1997). There were no significant differences between boys and girls in this sample regarding probability for success, which deviates from findings involving general samples of youth and young adults in both sexes (Lirgg, 1991; Lochbaum et al., 2019). This discrepancy may be due to cultural differences or the fact that this group was particularly homogeneous and therefore is expected to explain only minor proportions of performance (Baker et al., 2018). It is also possible that we did not find differences by sex because a specific type of motivation is required in this group of preselected players, and it is independent of sex (Sarmento et al., 2018).

Correlation analyses showed no significant correlations between the motivation variables and coach-reported performance. However, the relationship between probability of success and coach-reported performance was significantly as expected (partly confirming hypothesis 1). Due to the homogeneous population, it is conceivable that members of this population have similar motivations, even though it remains uncertain whether slight differences in motivation to achieve success or to avoid failure can explain variances in the performance of a high-performing young soccer population (Murr et al., 2018). Here, small margins and complex processes may be more important than individual variables for distinguishing between performances (Williams et al., 2020).

Similarly, to Elliot’s hierarchical model of motivation, the success motive predicts task-based and self-based approach goals and the motive to avoid failure predicted all three types of avoidance-based (task, self, and other) and other-based approach goals (confirming hypothesis 5). There is, however, no significant relationship between the success motive and other-based approach goals among the top soccer talents. Moreover, it appears that when players have a high probability of achieving success, they more frequently set other-based approach goals. This is in line with the results from the study by Van Yperen and Renkema (2008). It appears that the goal of outperforming others can arise from both a motive to avoid failure and a perception of having a high probability of success. In contrast, a high motive for success seems to contribute to players setting goals related to personal growth/learning (self-based approach) or to focusing on the task at hand (task-based approach). It further appears that a low probability of success contributes to high self- and other-based avoidance goals in players with a high motivation to avoid failure but higher task-based approach goals in those with a high motivation to seek success. However, a combination of low probability of success and high fear motivation can also lead to the adoption of more appropriate goals in the form of self- and other-based approach goals (confirming hypothesis 7).

Concerning self-regulated learning, the results from the process model indicated that the motive to achieve success and the probability of success were important predictors of planning, reflection/evaluation, and regulation of effort (confirming hypotheses 1 and 2). Toering et al. (2009) found that players with high reflection scores were 4.9 times more likely to play for the top clubs’ academy teams, and players with high effort scores were seven times more likely to play in an academy team, than those with low scores. Furthermore, Toering et al. (2012b) found in another study international players to score higher on reflection than national-level players did. The current study found that task-based and self-based approach goals predicted regulation of effort but not reflection and planning (partly confirming hypothesis 4). In addition, the mastery-approach goals mediated some of the correlation the motive to achieve success on the one hand and regulation of effort on the other hand (partly confirming hypothesis 8).

Some studies have shown no correlation between mastery goals and future performance (Figueiredo et al., 2009; Huijgen et al., 2014). Silva et al. (2010) found regional team players to be more oriented toward performance goals than local players, with no significant differences in mastery goals, and Kavussanu et al. (2011) found that elite team players score significantly higher on mastery goals than non-elite team players, with no differences in scores for performance goals. Meanwhile, Höner and Feichtinger (2016) found a significant positive correlation between mastery goals and performance level 4 years later in young soccer players. Van-Yperen and Duda (1999) found that improvements in performance during the soccer season corresponded to mastery goals. There is also a positive trend in our results for the relationship between approach goals and coach-reported performance, *r* = 0.7, 0.8, and 0.9, for self-, task-, and other-based approach goals, respectively (partly confirming hypothesis 3). It will be interesting to see whether the relationship between approach goals and performance will become stronger over time.

For avoidance paths, the data supported some of our original hypotheses. The motive to avoid failure predicted avoidance- and other-based approach goals (confirming hypothesis 5) but did not contribute to explaining self-regulation and performance (not confirming hypothesis 6). As noted, few studies have investigated the distinction between approach and avoidance goals within a soccer context. Nonetheless, studies in sports suggest that mastery and performance-approach goals are positively related to performance, while no significant relationship has been found between avoidance goals and performance (Van Yperen et al., 2014). However, for athletes under the age of 18 years, the results of a meta-analysis showed that the correlation between performance-approach goals and performance was not significant (Lochbaum and Gottardy, 2015). The discrepancy in results from earlier studies may be because performance goals in the Task and Ego Orientation in Sport Questionnaire (TEOSQ) do not distinguish between performance-approach and performance-avoidance. It is also possible that anxious players were already selected out in this preselected group.

The results from the moderation analysis showed that players with a high motive for success regulate effort, using metacognitive strategies (planning and reflection/evaluation) even when the likelihood of success is low (confirming hypothesis 7). However, players who score low on the motive for success rely on experiencing a relatively high probability of success to make optimal use of metacognitive strategies and regulate their effort. This is likely one of the reasons why the motive to achieve success has been shown to predict performance (Murr et al., 2018; Wachsmuth et al., 2023). However, high fear motivation also seems to predict regulation of effort when the likelihood of success is low. Perhaps this is caused by anxiety-driven form of perfectionism (Haraldsen et al., 2020). To examine whether this form of fear-driven regulation is effective over time, longitudinal studies are needed.

### Limitations and future studies

The sample size was large for a study of young national soccer team players. There are few studies on talented soccer girls’ motivation and self-regulated learning. In the present study, the sample included many girls, and we used well-tested instruments. Nevertheless, there are several possible limitations. The design limits the ability to confirm a causal relationship between the variables. Performance was only obtained from national team coaches. In future studies, we will collect register data on dropouts, playing time, and the level at which participants play as performance outcomes.

## Conclusion

In summary, this study aimed to investigate the motivational processes behind self-regulated learning and performance among soccer players eligible for the Norwegian national football team. The process models which examined achievement goals as mediators between probability of success and achievement motives on the one hand and performance and self-regulated learning on the other hand showed that task- and self-based approach goals mediated the relationship between the motive to achieve success and effort. However, that was the only outcome mediated by achievement goals. The results suggest that the direct effect of the motivation to seek success and the probability of success are more crucial for regulating effort and concentration and utilizing metacognitive strategies among potential national team talents in football. Scoring high on the motive to success appears to be of crucial importance for self-regulated learning in those who perceive a low probability of success. The data supported only some of our original hypotheses concerning avoidance paths, where the motive to avoid failure predicted avoidance- and other-based approach goals but did not contribute to explaining self-regulation and performance. In addition, a negative correlation was found between the motive to avoid failure and effort. Related to sex, girls were more motivated to avoid failure than boys, while both sexes achieved similar scores for football-specific self-regulated learning, probability of success, achievement goals, and motivation to achieve success. Future research should continue to expand the research on the impact of approach and avoidance goals and add on to the few empirical studies on the outcomes of the probability of success and achievement motives among talented young footballers who are eligible for national teams, and especially include girls.

## Data Availability

The raw data supporting the conclusions of this article will be made available by the authors, without undue reservation.
