# Supplementary material for: Self-regulation and performance among elite youth soccer players: the role of approach-avoidance motivation
Source: Front Psychol. 2024 Oct 14;15:1416931. doi: 10.3389/fpsyg.2024.1416931 (PMC11514363; doi:10.3389/fpsyg.2024.1416931)
Supplement: Supplementary file 1 [file Table_1.pdf]

Table 1 Supplementary.

Items and Factor loadings from confirmatory factor analysis of the Soccer-specific self-regulated learning scales (n = 192).

	Planning	Evaluation/ Reflection	Regulation of effort
1. Before practice tasks, I figure out what I need to do to accomplish specific goals (McCardle et al.'s, item 20)	.79		
3. Before practice exercises, I carefully think through the components of the exercise I am going to execute (McCardle et al.'s, item 2)	.57		
4. I plan how I will work on my strengths and weaknesses in advance of training sessions (new item)	80		
11. Before each practice session I plan my actions relative to the goal I want to attain during the practice session (Toering et al. 2013, Item 13)	80		
5. Each practice session I think back and evaluate whether I did the right things to become a better player (Toering et al. 2013, Item, 17)		.66	
2. After soccer training and matches I think about what I did right and wrong during the session (Toering et al. 2013, item 21)		.59	
7. Each practice session I keep track of my performance during practice, so that I can see which football skills I must improve (for example, technique, tactics) (Toering et al. 2013, item 19)		.72	
13. During each practice session I check whether I make progress in my football skills (Toering et al. 2013, item 2)		.51	
6. I usually keep working hard even when training tasks become difficult (McCardle et al., item 50)			.68
8. I don't give up at practice even if the task is hard McCardle et al., item 56)			.75
10. Even when I don't like a task during practice, I work hard (McCardle et al., item 39)			.55
